# Spinal NF-κB and Chemokine Ligand 5 Expression during Spinal Glial Cell Activation in a Neuropathic Pain Model

**DOI:** 10.1371/journal.pone.0115120

**Published:** 2015-01-30

**Authors:** Qin Yin, Qin Fan, Yu Zhao, Ming-Yue Cheng, He Liu, Jing Li, Fei-Fei Lu, Jin-Tai Jia, Wei Cheng, Chang-Dong Yan

**Affiliations:** 1 Xuzhou Medical College, Xuzhou, China; 2 Jiangsu Province Key Laboratory of Anesthesiology and Center for Pain Research and Treatment; Jiangsu Province Key Laboratory of Anesthesiology, Xuzhou, China; 3 Affiliated Hospital of Xuzhou Medical College, Xuzhou, China; 4 Affiliated Heping Hospital of Changzhi Medical College, Changzhi, China; Boston Children’s Hospital and Harvard Medical School, UNITED STATES

## Abstract

**Background:**

The NF-κB pathway and chemokine (C-C motif) ligand 5 (CCL5) are involved in pain modulation; however, the precise mechanisms of their interactions in chronic neuropathic pain have yet to be established.

**Methods:**

The present study examined the roles of spinal NF-κB and CCL5 in a neuropathic pain model after chronic constriction injury (CCI) surgery. CCI-induced pain facilitation was evaluated using the Plantar and von Frey tests. The changes in NF-κB and CCL5 expression were analyzed by immunohistochemistry and Western blot analyses.

**Results:**

Spinal NF-κB and CCL5 expression increased after CCI surgery. Repeated intrathecal infusions of pyrrolidine dithiocarbamate (PDTC, a NF-κB inhibitor) decreased CCL5 expression, inhibited the activation of microglia and astrocytes, and attenuated CCI-induced allodynia and hyperalgesia. Intrathecal injection of a CCL5-neutralizing antibody attenuated CCI-induced pain facilitation and also suppressed spinal glial cell activation after CCI surgery. However, the CCL5-neutralizing antibody did not affect NF-κB expression. Furthermore, selective glial inhibitors, minocycline and fluorocitrate, attenuated the hyperalgesia induced by intrathecal CCL5.

**Conclusions:**

The inhibition of spinal CCL5 expression may provide a new method to prevent and treat nerve injury-induced neuropathic pain.

## Introduction

Neuropathic pain is a therapeutic challenge and is often associated with peripheral nerve injury with characteristic pain facilitation. Previous studies have suggested that chemokines play an essential role in glial cell activation, inflammatory pain and neuropathic pain [1–3]. Glial selective inhibitors partially antagonize pain hypersensitivities and the up-regulation of chemokines in different pain models [4–9]. Nevertheless, the neuroimmune mechanisms that mediate glial cell activation in neuropathic pain are still unknown.

Chemokine (C-C motif) ligand 5 (also CCL5) is secreted by macrophages, platelets, and glial cells in the central nervous system (CNS) [10–13]. Furthermore, intracistemal injection of CCL5 remarkably increased the duration and amount of scratching in the itching model [14]. When the midbrain periaqueductal grey (PAG) receives a CCL5 injection, apparent hyperalgesia is observed [15]. These results highlight the significance of chemokines in the CNS [16]. Studies have previously demonstrated that CCL5 may play a role in different pain models in the spinal cord [17–21]. Activating the NF-κB pathway often promotes the activation of a series of genes and neurotransmitters, which leads to chemokine secretion and pain hypersensitivities [22, 23]. Intrathecal infusion of the NF-κB inhibitor (pyrrolidine dithiocarbamate, PDTC) delays and reverses pain facilitation in neuropathic pain [23–26].

However, the precise mechanisms of the NF-κB pathway and the interactions between NF-κB and CCL5 in chronic neuropathic pain have yet to be established. NF-κB inhibition may attenuate pain facilitation via CCL5 inhibition at the spinal level. We investigated the underlying mechanisms of the expression and inhibition of glial cell activation as well as NF-κB and CCL5 and their interactions in the spine in a neuropathic pain model following CCI surgery.

## Methods

### Experimental animal

Male SD rats (250–280 grams, 6–8 weeks) were housed in groups of 2 in clear plastic cages with solid floors covered with 3–6 cm of soft bedding (sawdust) and were maintained in controlled environments (21 ± 2°C; 60–70% relative humidity; 12 h dark/light cycles with ad libitum access to food and water). The rats were acclimatized for three days before any empirical procedures. All testing procedures were approved by the Animal Ethics Committee of Xuzhou Medical College. All experiments were conducted in compliance with the institutional guidelines.

### CCI surgery

A CCI-induced neuropathic pain model was established according to a previously described method [27]. Four chromic gut ligatures were loosely created around the left sciatic nerve after anesthesia (pentobarbital 50 mg/kg, i.p.). Sham-operated animals underwent the same surgical procedure, but no ligatures were placed around the nerve. The animals were allowed to recover for 72 hours to ensure the well-being of the rats after the CCI surgery. Only rats that exhibited a normal gait were included in the experiments.

### Intrathecal catheter

Lumbosacral intrathecal catheters were constructed and implanted as detailed in a previous study [28]. This method avoids pressure on the spine and the reactive ensheathment during surgery. The catheter was utilized to thread caudally from the cisterna magna after anesthesia (pentobarbital, 50 mg/kg, i.p.). The catheter locations were verified by visual inspection after the behavioral analysis. Only the data obtained from rats in which the distal ends of the catheter were located at the lumbo-sacral spinal level were analyzed.

### Drugs and peptides

Pyrrolidine dithiocarbamate (PDTC), minocycline and fluorocitrate were obtained from Sigma (St. Louis, MO, USA). The normal goat IgG, anti-CCL5 neutralizing antibody and recombinant rat CCL5 were purchased from R&D Systems (Minneapolis, MN, USA). Anti-rat CCL5, rabbit anti-rat NF-κB p65 and mouse anti-rat β-actin were obtained from Santa Cruz (Santa Cruz, CA, USA). Fluorescein isothiocyanate (FITC)-conjugated IgG and tetraethyl rhodamine isothiocyanate (Jackson Immunolab, West Grove, PA, USA), glial fibrillary acidic protein (GFAP, Millipore, Bedford, MA, USA), ionized calcium–binding adapter molecule 1 (Iba-1, Abcam), and neuronal specific nuclear protein (NeuN, neuronal marker, NOVUS) were purchased. The dosages of intrathecal drugs and peptides were chosen according to former studies [17, 29] and our preliminary tests.

### Von Frey test

The rats were placed on a 5 × 5 mm wire mesh grid floor, and testing was conducted blindly with respect to the group. Consistent with the Chaplan study [30], mechanical allodynia was observed by withdrawal responses using von Frey incitation after a 30-min accommodation. The von Frey filaments were inserted through the mesh floor bottom and were applied to the middle of the plantar surface of the hind paw with a weight of 4.0, 6.0, 8.0, 10.0, and 15.0 g (Stoelting, Wood Dale, IL, USA). The 50% paw withdrawal threshold was determined using Chaplan’s up-down method as previously described.

### Plantar Test

The rats were placed on top of a glass sheet and covered with a clear cage. After adapting for 30 minutes, thermal hyperalgesia was evaluated by withdrawal latency using the Plantar Test Analgesia Meter (BEM-410A, Chinese Academy of Medical Sciences Medical Research Institute of Biology). The radiant heat source was positioned under the glass sheet and applied to the plantar surface of the hind paw. The withdrawal latencies of the hind paws were measured five times at 5 min intervals. Data are presented as the mean latency of the last three stimulations [31]. A cut-off latency of 25 s was set for each measurement to avoid tissue damage.

### Western blot analysis

The left (ipsilateral to the CCI side) L4–5 spinal cord segments were collected for Western blot analyses. The total protein was extracted from the spinal segments, and 20 μg of extracts were separated by 10–15% SDS-PAGE and transferred to a PVDF membrane. The membrane was blocked with 5% nonfat dry milk and incubated with mouse anti-rat CCL5 (1:100) and rabbit anti-rat NF-κB (1:1000) or mouse anti-rat β-actin (1:1000) primary antibodies. The membrane was washed and incubated with alkaline phosphatase (ALP)-conjugated goat anti-rabbit or goat anti-mouse secondary antibody and treated with the NBT/BCIP Western blotting substrate (Promega Corporation, Madison, WI, USA). All Western blots were performed at least three times, and the data were consistent among the experiments. A previous method was utilized to calculate the density of the band area [32]. An equivalent-sized square was drawn to quantify the density around each band, and the background surrounding the band was subtracted. β-actin expression was utilized as an internal control, and the protein level was standardized to the β-actin level.

### Immunohistochemistry

The L4–5 spinal segments were post-fixed in fixative for 24 h at 4°C and immersed in 30% sucrose in PBS for 24–48 h at 4°C for cryoprotection. A frozen longitudinal slice (20–30 μm) was prepared. The section was blocked with 10% donkey serum in PBS and was incubated with the mouse anti-rat CCL5 (1:50) and rabbit anti-rat NF-κB (1:100) antibodies overnight at 4°C. The antibodies against the proteins of spinal cord cells include NeuN (1:600), GFAP (1:300), and Iba-1 (1:400). The sections were incubated in specific secondary antibodies that were conjugated with FITC-conjugated IgG (1:200) or tetraethyl rhodamine isothiocyanate (1:200) for 120 min at 4°C and then washed in PBS. The primary antibody was omitted in the negative control. All sections were cover-slipped with a mixture of 50% glycerin in 0.01 M PBS and then viewed under a Leica fluorescence microscope. The images were captured with a CCD spot camera. The cell counts may not sufficiently reflect activation due to the complex morphology of the neurons and gliocytes and the immunoreactive staining associated with cell bodies and their processes. Therefore, the optical density of the immunoreactive staining was measured with the Leica Qwin 500 image analysis system (Germany). The relative density of the images was determined by subtracting the background density in each image. Six spinal L4–5 sections were randomly selected from each animal for densitometric analysis to obtain the mean density for each animal.

### Statistical analysis

The data in the results section are presented as the mean ± standard error (S.E.M.). All experiments were performed blindly. The Tukey’s post-hoc test in one-way ANOVA was utilized to perform multiple comparisons between all groups tested. The Student–Newman–Keuls post-hoc test and repeated measures ANOVA in two-way ANOVA were utilized to analyze the post-drug time course measures in the behavioral tests. Statistical significance was established at *P* < 0.05.

## Results

### PDTC delayed and attenuated mechanical allodynia and thermal hyperalgesia induced by CCI surgery

**Figure 1:**
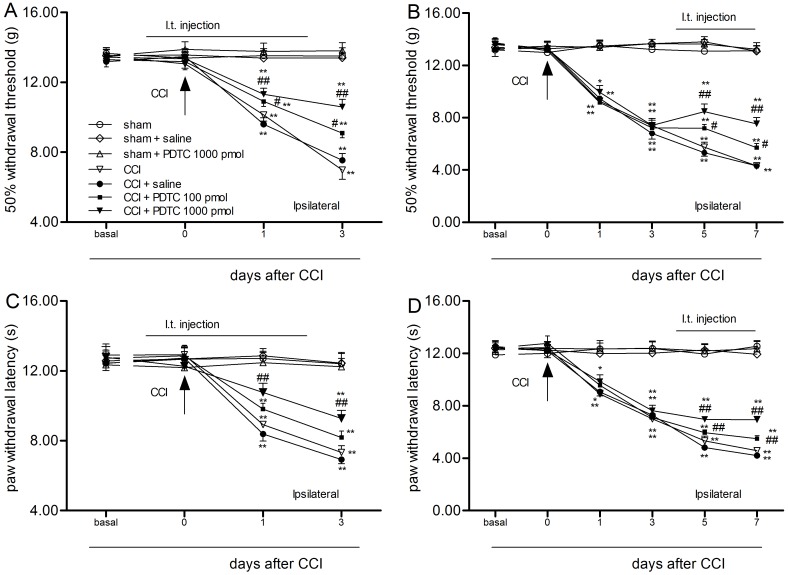
Intrathecal use of PDTC on days 0–2 and days 4–7 delayed and attenuated tactile allodynia (A, B) and thermal hypersensitivity (C, D) of the ipsilateral limb. *P < 0.05, **P < 0.01 vs. day 0; ^#^ *P* < 0.05, ^##^ *P* < 0.01 vs. the CCI + saline group (mean ± S.E.M., n = 8).

Repeated intrathecal use of PDTC (1000 pmol/d) did not change the paw withdrawal threshold (WT) or paw withdrawal latency (WL) in the sham + PDTC 1000 pmol/d group (ANOVA, *P >* 0.05). Compared with the rats in the CCI + saline group, the WT and WL of the CCI + PDTC group (100 pmol/d and 1000 pmol/d, on days 0–2 or day 4–7) were dose-dependently increased (two-way ANOVA, *P* < 0.01) (Fig. 1).

### PDTC suppressed the CCI-induced glial cell activation and NF-κB and CCL5 expression in the spinal segments

**Figure 2:**
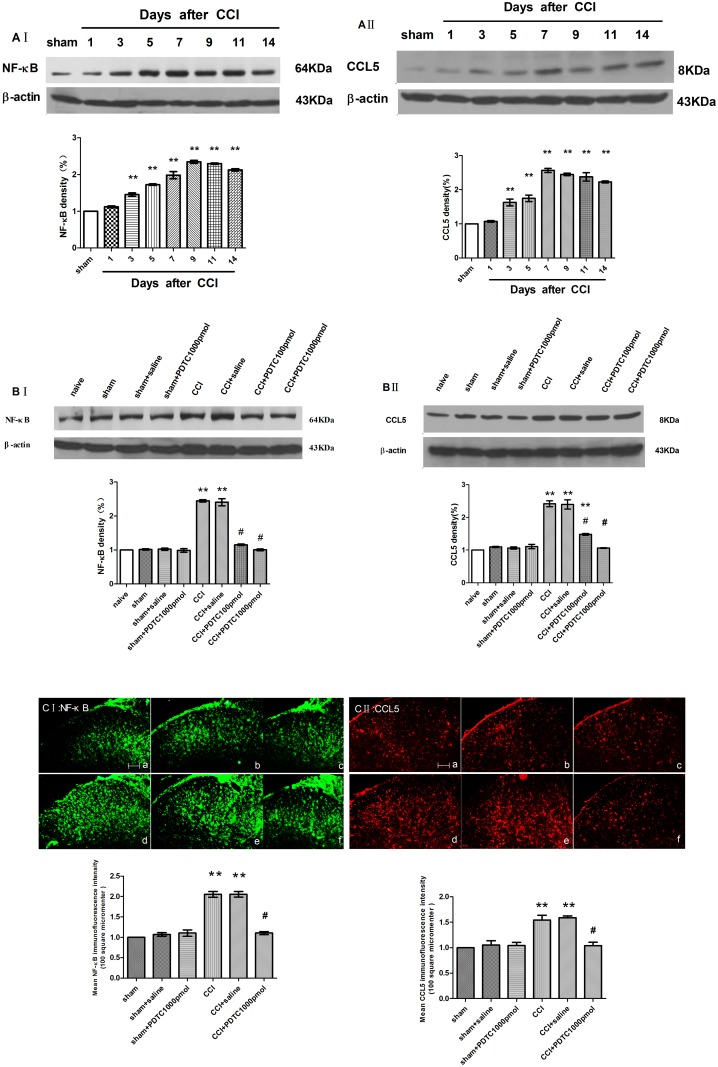
Spinal NF-κB and CCL5 expression after CCI surgery and intrathecal use of PDTC in the ipsilateral L4–5 spinal cord (mean ± S.E.M., n = 3). Time-course of spinal NF-κB and CCL5 expression after CCI surgery. *P < 0.05, **P < 0.01 vs. the sham group (AⅠ, Ⅱ). Intrathecal administration of PDTC 4–7 days following CCI surgery inhibited the increase in NF-κB and CCL5 expression (western-blot (B Ⅰ, Ⅱ) and immunohistochemistry (C Ⅰ, Ⅱ)). The ipsilateral L4–5 spinal cord segments were collected on day 7 after surgery. **P < 0.01 vs. the sham group; ^#^ *P* < 0.01 vs. the CCI + saline group. (a) Sham group; (b) Sham + saline group; (c) Sham + PDTC, 1000 pmol group; (d) CCI group; and (e) CCI + saline group; (f) CCI + PDTC, 1000 pmol group. Scale bar = 100 μm.

The ipsilateral L4–5 spinal cord segments were collected on day 7 after CCI surgery and were examined. Western blot analysis indicated that the NF-κB and CCL5 expression remarkably increased in the ipsilateral spinal segments compared with the sham group (ANOVA, *P* < 0.01) (Fig. 2A).

According to the western blot (Fig. 2B) and immunohistochemistry (Fig. 2C) analysis, PDTC attenuated the CCI-induced changes in NF-κB and CCL5 expression, as demonstrated by the decreased intensity of NF-κB and CCL5 (ANOVA, *P* < 0.01).

**Figure 3:**
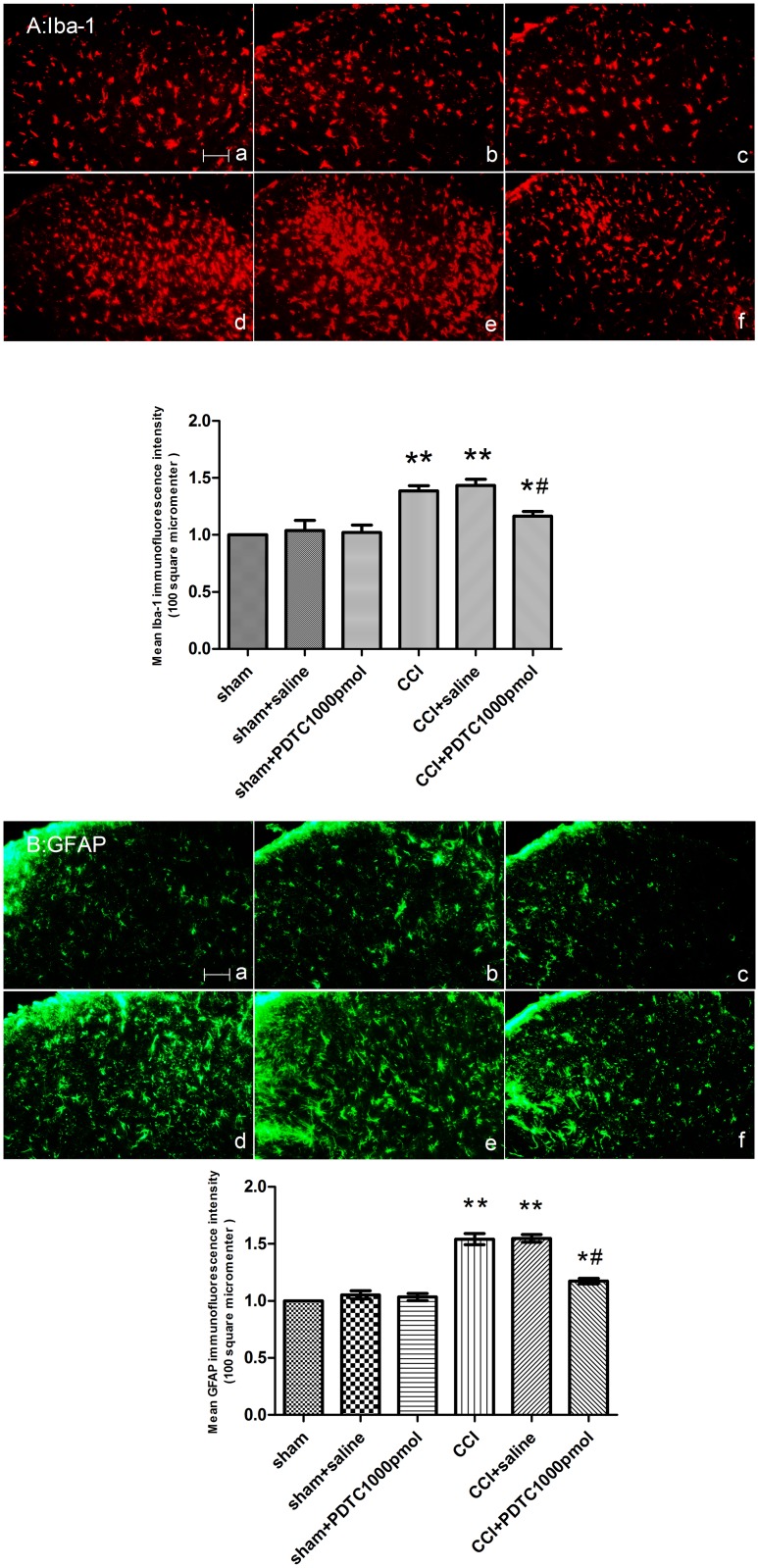
Intrathecal administration of PDTC 4–7 days following CCI surgery attenuated the CCI-induced glial cell activation. The ipsilateral L4–5 spinal cord segments were collected on day 7 after surgery (A: Iba-1, B: GFAP). *P < 0.05, **P < 0.01 vs. the sham group; ^#^ *P* < 0.01 vs. the CCI + saline group. (a) Sham group; (b) Sham + saline group; (c) Sham + PDTC, 1000 pmol group; (d) CCI group; and (e) CCI + saline group; (f) CCI + PDTC, 1000 pmol group; Scale bar = 100 μm (mean ± S.E.M., n = 3).

PDTC attenuated the up-regulation of spinal GFAP and Iba-1 following CCI surgery as shown by the reduced intensity of GFAP and Iba-1 staining (ANOVA, *P* < 0.01, comparing the CCI + saline and CCI + PDTC 1000 pmol/d groups) (Fig. 3).

### CCL5-neutralizing antibodies delayed and attenuated CCI-induced allodynia and hyperalgesia

**Figure 4:**
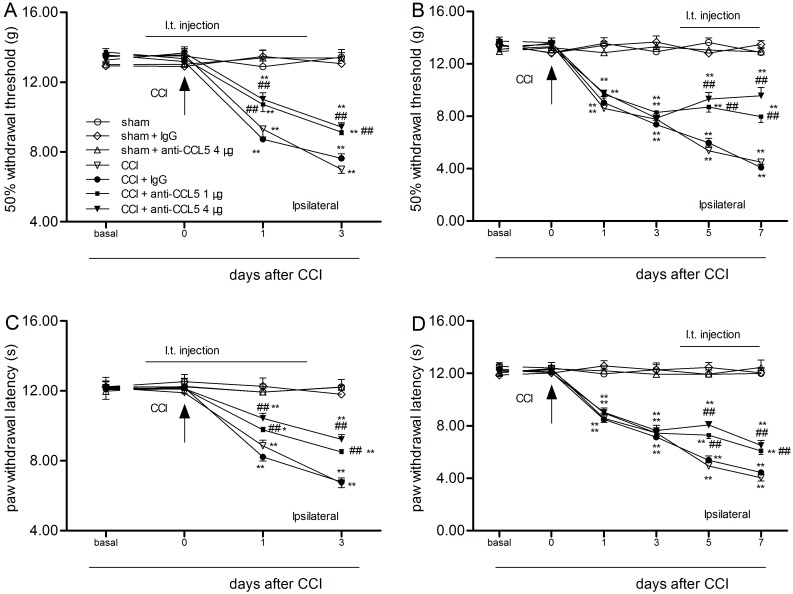
Intrathecal administration of the CCL5-neutralizing antibody on days 0–2 and days 4–7 delayed and attenuated tactile allodynia (A, B) and thermal hypersensitivity (C, D) of the ipsilateral limb. *P < 0.05, **P < 0.01 vs. day 0; ^#^ *P* < 0.05, ^##^ *P* < 0.01 vs. the CCI + control IgG group (mean ± S.E.M., n = 8).

Repeated intrathecal infusions of the CCL5-neutralizing antibody did not change the WT or WL in the sham + CCL5-neutralizing antibody group (ANOVA, *P >* 0.05). Compared with the animals in the CCI + control IgG group, the WT and WL of the CCI + CCL5-neutralizing antibody group (1 µg/d and 4 µg/d; on days 0–2 or day 4–7) were significantly increased (two-way ANOVA, *P* < 0.01) (Fig. 4).

### The CCL5-neutralizing antibody attenuated the CCI-induced glial cell activation but not NF-κB expression

**Figure 5:**
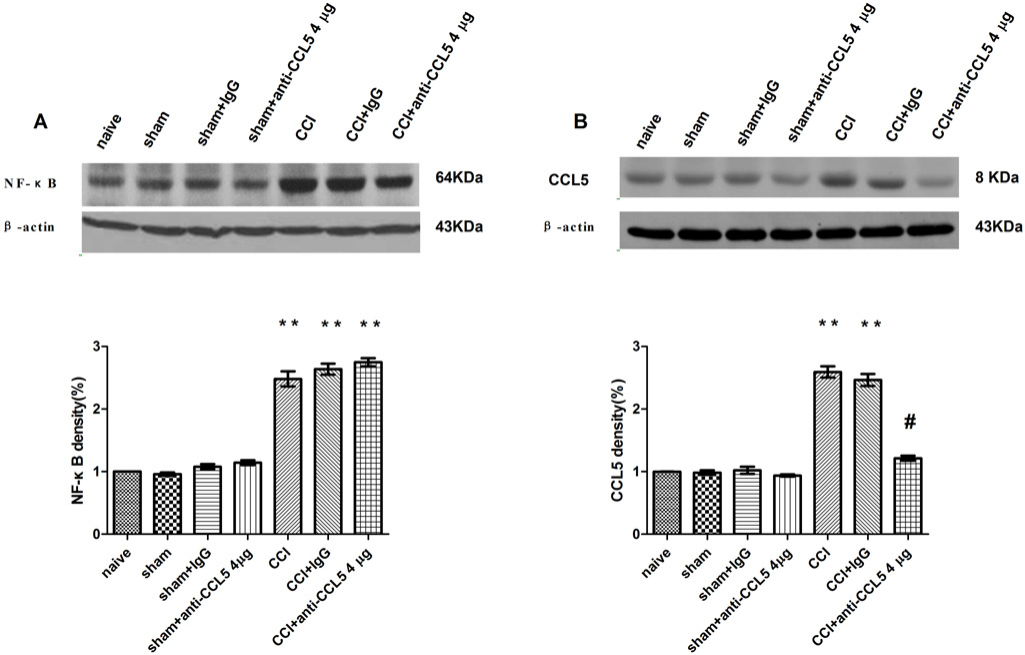
Intrathecal administration of the CCL5-neutralizing antibody on days 4–7 after CCI surgery attenuated the increase in CCL5 expression but not the CCI-induced NF-κB changes in the ipsilateral L4–5 spinal cord. **P < 0.01 vs. the sham group; ^#^ *P* < 0.01 vs. the CCI + control IgG group (mean ± S.E.M., n = 3).

In contrast to the CCI + control IgG group, the CCL5-neutralizing antibody (4 µg/d) attenuated the increase in CCL5 expression (ANOVA, *P* < 0.01) but did not affect NF-κB expression (ANOVA, *P >* 0.05, Fig. 5).

**Figure 6:**
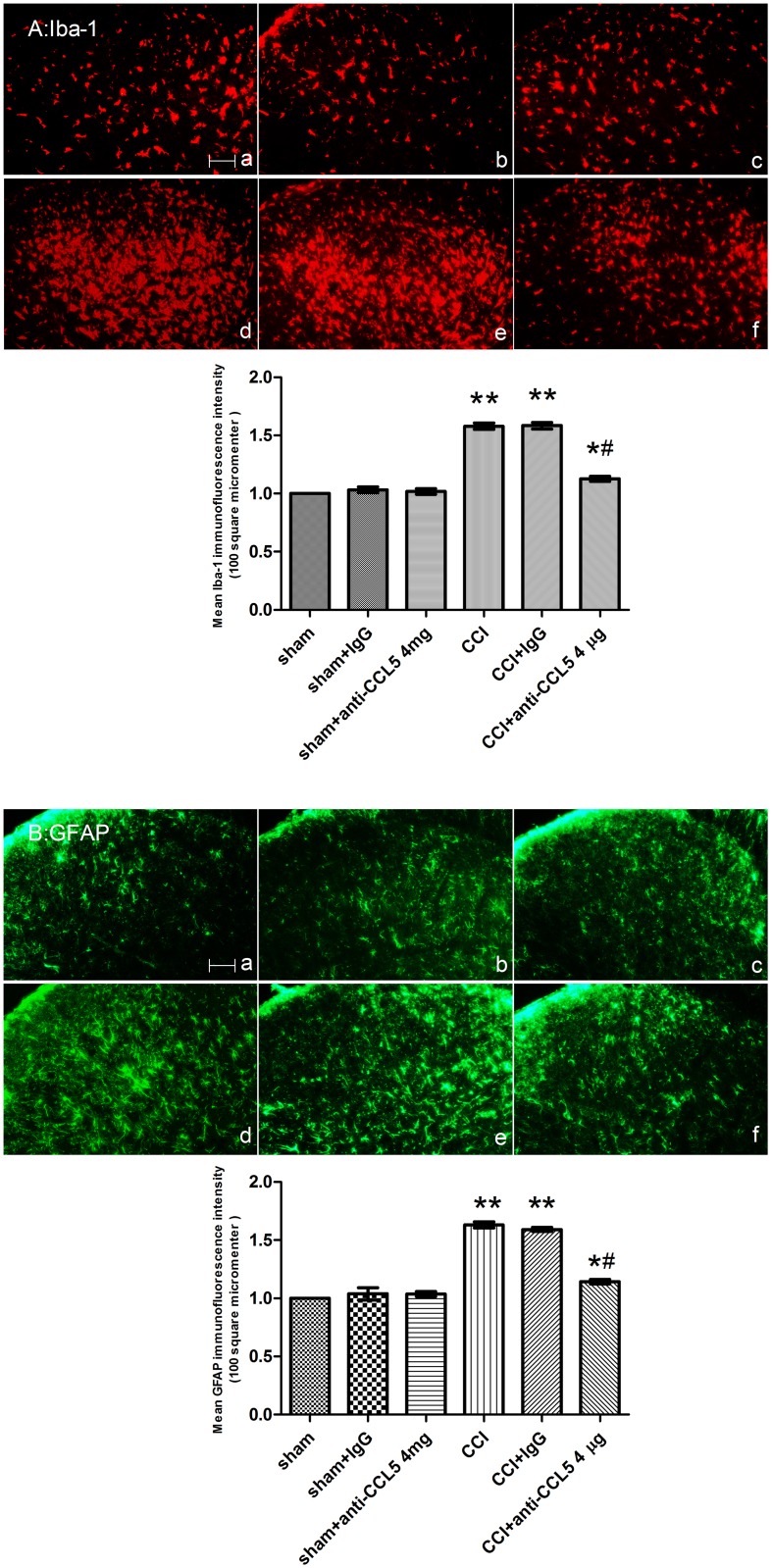
Intrathecal injections of the CCL5-neutralizing antibody (4 µg) on days 4–7 after CCI surgery blocked the CCI-induced glial cell activation in the ipsilateral L4–5 spinal cord (A: Iba-1, B: GFAP). (a) Sham group; (b) Sham + control IgG group; (c) Sham + CCL5-neutralizing antibody, 4 µg group; (d) CCI group; (e) CCI + control IgG group; (f) CCI + CCL5-neutralizing antibody, 4 µg group (mean ± S.E.M., n = 3). *P < 0.05, **P < 0.01 vs. the sham group; ^#^ *P* < 0.01 vs. the CCI + control IgG group. Scale bar = 100 μm. (mean ± S.E.M., n = 3).

The mean optical densities of spinal Iba-1 and GFAP immunoreactivity were greatly increased in the CCI rats compared with the sham group. Pre-administration of control IgG did not alter the CCI-induced glial cell activation. The CCL5-neutralizing antibody (4 µg/d) suppressed the activation of spinal microglia and astrocytes caused by the CCI surgery, as demonstrated by the decreased mean optical density of the GFAP and Iba-1 (ANOVA, *P* < 0.01) (Fig. 6).

### Double immunofluorescence of NF-κB and CCL5 with microglia, astrocytes, and neurons

**Figure 7:**
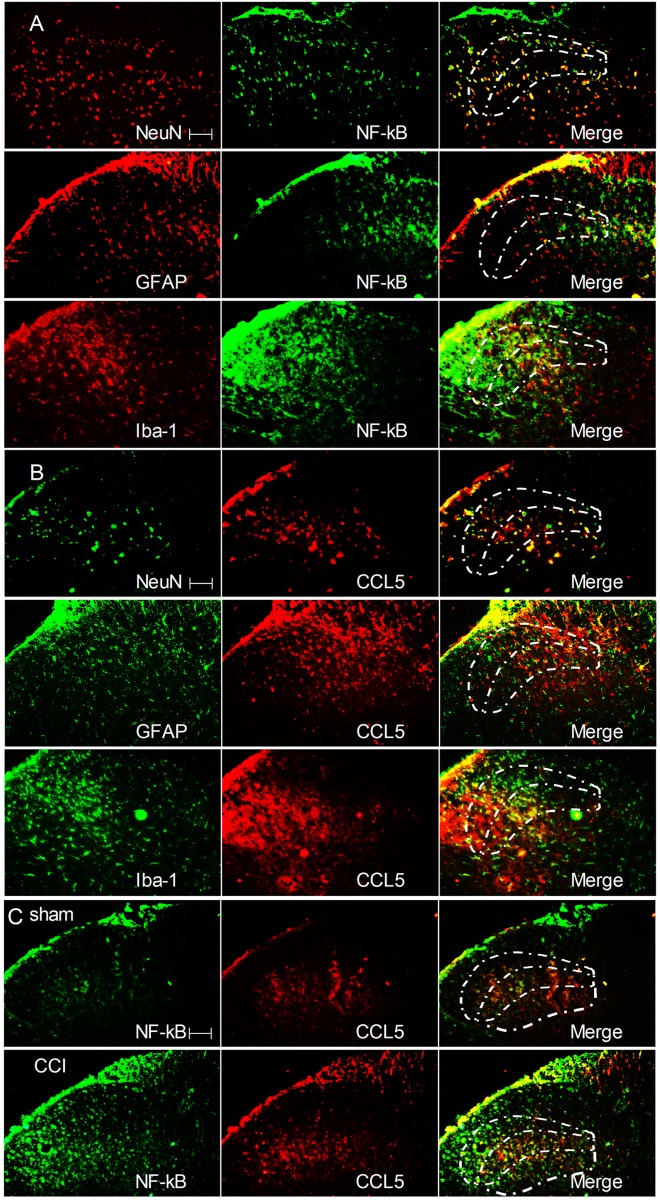
Double immunofluorescence indicates that NF-κB and CCL5 were co-localized with microglia, astrocytes, and neurons in the ipsilateral L4–5 spinal cord on day 7 after CCI. NF-κB (green) colocalizes with Iba-1, GFAP, and NeuN (red) in laminae II–III of the superficial dorsal horn (A). CCL5 (red) co-localizes with Iba-1, GFAP, and NeuN (green) in the medial superficial dorsal horn (laminae II–III) (B). NF-κB (green) co-localizes with CCL5 (red) in laminae II–IV of the superficial dorsal horn following CCI on day 7 in the sham and CCI groups in the ipsilateral L4–5 spinal cord (C). Two single stained images (yellow) were merged. Scale = 100 μm.

Dual labeling indicates that the CCL5-IR and NF-κB-IR cells represented neurons, microglia and astrocytic cells, as these cell types also co-expressed NeuN, Iba-1 and GFAP in the ipsilateral L4–5 spinal cord on day 7 after CCI surgery. Dual staining also indicates that NF-κB was co-localized with CCL5 in the medial ipsilateral dorsal horn (Fig. 7).

### Minocycline or fluorocitrate attenuated the CCL5-induced hyperalgesia according to the Hargreaves test

**Figure 8:**
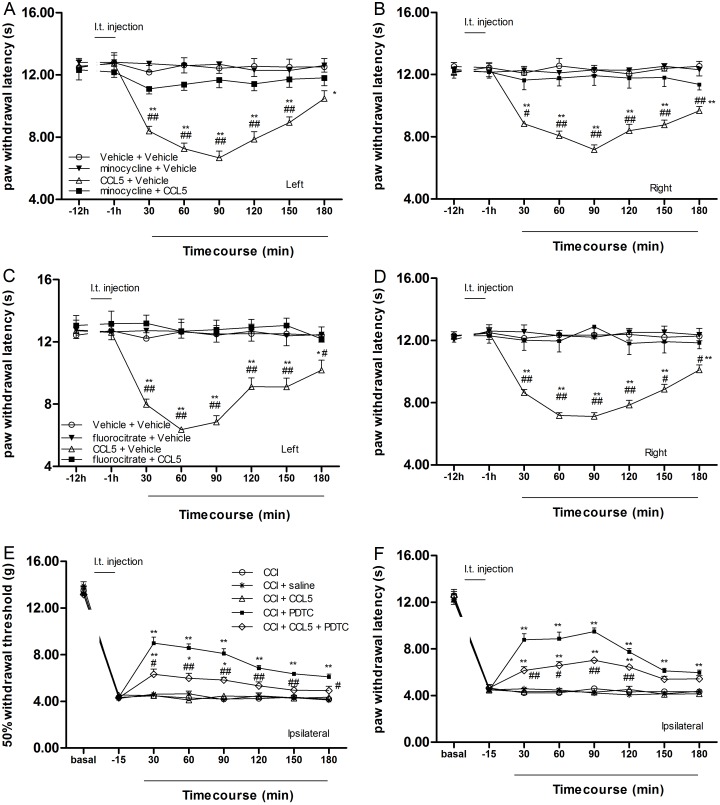
Minocycline or fluorocitrate attenuates the CCL5-induced hyperalgesia, and CCL5 attenuated the analgesic effects of PDTC. Administration of minocycline or fluorocitrate attenuated the hyperalgesia induced by CCL5 in Hargreaves test. In the left and right limbs, the rats exhibited marked thermal hypersensitivity following intrathecal administration of CCL5. Pretreatment with minocycline or fluorocitrate prevented pain in response to intrathecal injection of CCL5. **P < 0.01 vs. baseline (-1 h); ^#^ *P* < 0.05, ^##^ *P* < 0.01 vs. minocycline/fluorocitrate + CCL5 group (mean ± S.E.M., n = 8) (A-D). The analgesic effects of PDTC were partially antagonized by CCL5 (0.2 µg, i.t.) 15 min before intrathecal administration of PDTC. *P < 0.05, **P < 0.01 vs. -15min, ^#^ *P* < 0.05, ^##^ *P* < 0.01 vs. CCI + PDTC 1000 pmol/d (mean ± S.E.M., n = 8) (E, F).

Treatment with minocycline (Fig. 8A, B), fluorocitrate (Fig. 8C, D) or the vehicle did not affect the WL compared with the baseline values (ANOVA, *P >* 0.05). Intrathecal infusions of CCL5 produced obvious hyperalgesia (CCL5 main effect), and treatment with minocycline or fluorocitrate blocked the CCL5-induced hyperalgesia (two-way ANOVA, *P* < 0.01).

### Intrathecal CCL5 partially attenuated the analgesic effects of PDTC in CCI rats

No effects were observed with the intrathecal administration of normal saline or CCL5 (0.2 μg) (ANOVA, *P >* 0.05). The WT and WL in the CCI + PDTC group were significantly increased compared with the CCI + saline group (ANOVA, *P* < 0.01). The effects of PDTC were attenuated by CCL5 (0.2 µg, i.t., 15 min before PDTC) (two-way ANOVA, *P* < 0.01) (Fig. 8E, F).

## Discussion

In the present study, we found that the increase in spinal CCL5 after CCI surgery occurred in parallel with the glial cell activation of the spinal cords and the development of neuropathic pain. Intrathecal administration of CCL5-neutralizing antibody delayed and attenuated the initiation of pain hypersensitivities following CCI surgery, and the CCL5-neutralizing antibody inhibited CCI-induced glia activation in the spinal cords. Inhibition of microglia activation or astrocyte activation relieved the intrathecal CCL5-induced pain facilitation. Therefore, CCL5-induced pain facilitation was regulated by microglia or astrocyte activation in the spine.

Several studies have demonstrated that glial cells (microglia and astrocytes) and neurons secrete CCL5. The involvement of CCL5 [14, 15, 17] and its receptors (including CCR1 [33–35], CCR3 and CCR5 [18–20, 36, 37, 38]) has also been observed in different pain models [39–41]. CCL5 plays a specific role in the complex chemical interaction between glial cells and neurons and helps maintain CNS homeostasis, as may other chemokines. After CCL5-induced activation, microglia secretes glial-excitatory transmitters, leading to astrocytic activation. Varieties of neuro- and glial-excitatory transmitters are secreted by activated microglia and astrocytes [42–46], which may lead to the initiation and maintenance of neuropathic pain. Therefore, the prevention of CCL5 and glial cell activation blocks the occurrence and development of CCI-induced pain hypersensitivities.

Furthermore, we also showed that the intrathecal administration of PDTC attenuated the CCI-induced glial cell activation and increases in NF-κB and CCL5 expression. The intrathecal injection of CCL5 partially attenuated the analgesic effects of PDTC in CCI rats, suggesting that the decrease in CCL5 expression and glial cell activation may be involved in the anti-nociceptive mechanisms of PDTC’s analgesic effects. Our data have extended the results of previously published studies [22, 25, 26, 29, 47, 48] by showing that PDTC produces analgesic effects in chronic models via the inhibition of spinal NF-κB and CCL5 expression and the activation of spinal glia and by indicating that the NF-κB-CCL5 pathway mediates neuropathic pain through the regulation of CCL5 expression.

In various types of pain, NF-κB mediates immune and inflammatory responses via the regulation of genes that can encode proinflammatory cytokines, adhesion molecules, and chemokines in the spinal cords [23, 26]. Microglia activation may first lead to a series of spinal immune responses. In addition, NF-κB inhibition by PDTC reduces the expression of spinal CX3CR1 in a CCI model [29] and the expression of spinal COX-2 in the SNL model. The changes in TNF-α, IL-6 and interleukin (IL)-1A in the CSF were remarkably related to the changes in NF-κB in the gp120-injected rats [25]. The negative mediation of NF-κB on these pro-inflammatory factors and chemokines may explain the analgesic effects of PDTC. The data presented above may explain why PDTC suppressed the up-regulation of CCL5 and glia activation after CCI surgery and why intrathecal administration of CCL5 partially attenuated the anti-nociceptive effects of PDTC in CCI rats. Our data also indicate that NF-κB may not be the sole neurotransmitter involved in spinal glial cell activation (microglia or astrocyte activation) in the CCI rats.

In conclusion, our data provide new evidence supporting the hypothesis that spinal NF-κB and CCL5 play a role in the induction and development of neuropathic pain through glial cell activation. Inhibition of spinal CCL5 may offer a novel method to prevent and treat nerve injury-induced neuropathic pain.

## References

[1] Gosselin RD, Dansereau MA, Pohl M, Kitabgi P, Beaudet N, et al (2008) Chemokine network in the nervous system: a new target for pain relief. Curr Med Chem 15: 2866–2875. 10.2174/092986708786242822 18991641

[2] Hui J, Zhang ZJ, Zhang X, Shen Y, Gao YJ (2013) Repetitive hyperbaric oxygen treatment attenuates complete Freund’s adjuvant-induced pain and reduces glia-mediated neuroinflammation in the spinal cord. J Pain 14: 747–758. 10.1016/j.jpain.2013.02.003 23680474

[3] Ji RR, Berta T, Nedergaard M (2013) Glia and pain: is chronic pain a gliopathy? Pain 154 Suppl 1: S10–S28. 10.1016/j.pain.2013.06.022 23792284 PMC3858488

[4] Guasti L, Richardson D, Jhaveri M, Eldeeb K, Barrett D, et al (2009) Minocycline treatment inhibits microglial activation and alters spinal levels of endocannabinoids in a rat model of neuropathic pain. Mol Pain 5: 35 10.1186/1744-8069-5-35 19570201 PMC2719614

[5] Lin CS, Tsaur ML, Chen CC, Wang TY, Lin CF, et al (2007) Chronic intrathecal infusion of minocycline prevents the development of spinal-nerve ligation-induced pain in rats. Reg Anesth Pain Med 32: 209–216. 10.1016/j.rapm.2007.01.005 17543815

[6] Mei XP, Chen L, Wang W, Wu D, Wang LY, et al (2013) Combination of tramadol with minocycline exerted synergistic effects on a rat model of nerve injury-induced neuropathic pain. Neurosignals 21: 184–196. 10.1159/000338049 22964800

[7] Mei XP, Xu H, Xie C, Ren J, Zhou Y, et al (2011) Post-injury administration of minocycline: an effective treatment for nerve-injury induced neuropathic pain. Neurosci Res 70: 305–312. 10.1016/j.neures.2011.03.012 21515316

[8] Pu S, Xu Y, Du D, Yang M, Zhang X, et al (2013) Minocycline attenuates mechanical allodynia and expression of spinal NMDA receptor 1 subunit in rat neuropathic pain model. J Physiol Biochem 69: 349–357. 10.1007/s13105-012-0217-4 23111678

[9] Zhang X, Xu Y, Wang J, Zhou Q, Pu S, et al (2012) The effect of intrathecal administration of glial activation inhibitors on dorsal horn BDNF overexpression and hind paw mechanical allodynia in spinal nerve ligated rats. J Neural Transm 119: 329–336. 10.1007/s00702-011-0713-7 21901501

[10] Cocchi F, DeVico AL, Garzino-Demo A, Arya SK, Gallo RC, et al (1995) Identification of RANTES, MIP-1 alpha, and MIP-1 beta as the major HIV-suppressive factors produced by CD8+ T cells. Science 270: 1811–1815. 10.1126/science.270.5243.1811 8525373

[11] Keswani SC, Polley M, Pardo CA, Griffin JW, McArthur JC, et al (2003) Schwann cell chemokine receptors mediate HIV-1 gp120 toxicity to sensory neurons. Ann Neurol 54: 287–296. 10.1002/ana.10645 12953261

[12] Kraaijeveld AO, de Jager SC, de Jager WJ, Prakken BJ, McColl SR, et al (2007) CC chemokine ligand-5 (CCL5/RANTES) and CC chemokine ligand-18 (CCL18/PARC) are specific markers of refractory unstable angina pectoris and are transiently raised during severe ischemic symptoms. Circulation 116: 1931–1941. 10.1161/CIRCULATIONAHA.107.706986 17909104

[13] Wu H, Ghosh S, Perrard XD, Feng L, Garcia GE, et al (2007) T-cell accumulation and regulated on activation, normal T cell expressed and secreted upregulation in adipose tissue in obesity. Circulation 115: 1029–1038. 10.1161/CIRCULATIONAHA.106.638379 17296858

[14] Ahn DK, Lee KR, Lee HJ, Kim SK, Choi HS, et al (2005) Intracisternal administration of chemokines facilitated formalin-induced behavioral responses in the orofacial area of freely moving rats. Brain Res Bull 66: 50–58. 10.1016/j.brainresbull.2005.03.015 15925144

[15] Benamar K, Geller EB, Adler MW (2008) Elevated level of the proinflammatory chemokine, RANTES/CCL5, in the periaqueductal grey causes hyperalgesia in rats. Eur J Pharmacol 592: 93–95. 10.1016/j.ejphar.2008.07.009 18656466

[16] Lunzer MM, Yekkirala A, Hebbel RP, Portoghese PS (2007) Naloxone acts as a potent analgesic in transgenic mouse models of sickle cell anemia. Proc Natl Acad Sci U S A 104: 6061–6065. 10.1073/pnas.0700295104 17389363 PMC1851616

[17] Hang LH, Shao DH, Chen Z, Chen YF, Shu WW, et al (2013) Involvement of Spinal CC Chemokine Ligand 5 in the Development of Bone Cancer Pain in Rats. Basic Clin Pharmacol Toxicol. 10.1111/bcpt.12099 23773283

[18] Liou JT, Mao CC, Ching-Wah SD, Liu FC, Lai YS, et al (2013) Peritoneal administration of Met-RANTES attenuates inflammatory and nociceptive responses in a murine neuropathic pain model. J Pain 14: 24–35. 10.1016/j.jpain.2012.09.015 23183003

[19] Liou JT, Lee CM, Day YJ (2013) The immune aspect in neuropathic pain: role of chemokines. Acta Anaesthesiol Taiwan 51: 127–132. 10.1016/j.aat.2013.08.006 24148742

[20] Liou JT, Yuan HB, Mao CC, Lai YS, Day YJ (2012) Absence of C-C motif chemokine ligand 5 in mice leads to decreased local macrophage recruitment and behavioral hypersensitivity in a murine neuropathic pain model. Pain 153: 1283–1291. 10.1016/j.pain.2012.03.008 22494919

[21] Oh SB, Tran PB, Gillard SE, Hurley RW, Hammond DL, et al (2001) Chemokines and glycoprotein120 produce pain hypersensitivity by directly exciting primary nociceptive neurons. J Neurosci 21: 5027–5035. 11438578 10.1523/JNEUROSCI.21-14-05027.2001 PMC6762869

[22] Sun T, Song WG, Fu ZJ, Liu ZH, Liu YM, et al (2006) Alleviation of neuropathic pain by intrathecal injection of antisense oligonucleotides to p65 subunit of NF-kappaB. Br J Anaesth 97: 553–558. 10.1093/bja/ael209 16885169

[23] Wu LC, Goettl VM, Madiai F, Hackshaw KV, Hussain SR (2006) Reciprocal regulation of nuclear factor kappa B and its inhibitor ZAS3 after peripheral nerve injury. BMC Neurosci 7: 4 10.1186/1471-2202-7-4 16409637 PMC1361774

[24] Laughlin TM, Bethea JR, Yezierski RP, Wilcox GL (2000) Cytokine involvement in dynorphin-induced allodynia. Pain 84: 159–167. 10.1016/S0304-3959(99)00195-5 10666520

[25] Ledeboer A, Gamanos M, Lai W, Martin D, Maier SF, et al (2005) Involvement of spinal cord nuclear factor kappaB activation in rat models of proinflammatory cytokine-mediated pain facilitation. Eur J Neurosci 22: 1977–1986. 10.1111/j.1460-9568.2005.04379.x 16262636

[26] Wei XH, Zang Y, Wu CY, Xu JT, Xin WJ, et al (2007) Peri-sciatic administration of recombinant rat TNF-alpha induces mechanical allodynia via upregulation of TNF-alpha in dorsal root ganglia and in spinal dorsal horn: the role of NF-kappa B pathway. Exp Neurol 205: 471–484. 10.1016/j.expneurol.2007.03.012 17459378

[27] Bennett GJ, Xie YK (1988) A peripheral mononeuropathy in rat that produces disorders of pain sensation like those seen in man. Pain 33: 87–107. 10.1016/0304-3959(88)90209-6 2837713

[28] Milligan ED, Hinde JL, Mehmert KK, Maier SF, Watkins LR (1999) A method for increasing the viability of the external portion of lumbar catheters placed in the spinal subarachnoid space of rats. J Neurosci Methods 90: 81–86. 10.1016/S0165-0270(99)00075-8 10517276

[29] Pan YD, Guo QL, Wang E, Ye Z, He ZH, et al (2010) Intrathecal infusion of pyrrolidine dithiocarbamate for the prevention and reversal of neuropathic pain in rats using a sciatic chronic constriction injury model. Reg Anesth Pain Med 35: 231–237. 10.1097/AAP.0b013e3181df245b 20921832

[30] Chaplan SR, Bach FW, Pogrel JW, Chung JM, Yaksh TL (1994) Quantitative assessment of tactile allodynia in the rat paw. J Neurosci Methods 53: 55–63. 10.1016/0165-0270(94)90144-9 7990513

[31] Hargreaves K, Dubner R, Brown F, Flores C, Joris J (1988) A new and sensitive method for measuring thermal nociception in cutaneous hyperalgesia. Pain 32: 77–88. 10.1016/0304-3959(88)90026-7 3340425

[32] Zhuang ZY, Gerner P, Woolf CJ, Ji RR (2005) ERK is sequentially activated in neurons, microglia, and astrocytes by spinal nerve ligation and contributes to mechanical allodynia in this neuropathic pain model. Pain 114: 149–159. 10.1016/j.pain.2004.12.022 15733640

[33] Kiguchi N, Kobayashi Y, Maeda T, Saika F, Kishioka S (2010) CC-chemokine MIP-1alpha in the spinal cord contributes to nerve injury-induced neuropathic pain. Neurosci Lett 484: 17–21. 10.1016/j.neulet.2010.07.085 20692319

[34] Knerlich-Lukoschus F, von der Ropp-Brenner B, Lucius R, Mehdorn HM, Held-Feindt J (2011) Spatiotemporal CCR1, CCL3(MIP-1alpha), CXCR4, CXCL12(SDF-1alpha) expression patterns in a rat spinal cord injury model of posttraumatic neuropathic pain. J Neurosurg Spine 14: 583–597. 21332278 10.3171/2010.12.SPINE10480

[35] Pevida M, Lastra A, Meana A, Hidalgo A, Baamonde A, et al (2014) The chemokine CCL5 induces CCR1-mediated hyperalgesia in mice inoculated with NCTC 2472 tumoral cells. Neuroscience 259: 113–125. 10.1016/j.neuroscience.2013.11.055 24316469

[36] White FA, Wilson NM (2008) Chemokines as pain mediators and modulators. Curr Opin Anaesthesiol 21: 580–585. 10.1097/ACO.0b013e32830eb69d 18784482 PMC2702665

[37] Tokami H, Ago T, Sugimori H, Kuroda J, Awano H, et al (2013) RANTES has a potential to play a neuroprotective role in an autocrine/paracrine manner after ischemic stroke. Brain Res 1517: 122–132. 10.1016/j.brainres.2013.04.022 23602964

[38] Saika F, Kiguchi N, Kobayashi Y, Fukazawa Y, Kishioka S (2012) CC-chemokine ligand 4/macrophage inflammatory protein-1beta participates in the induction of neuropathic pain after peripheral nerve injury. Eur J Pain 16: 1271–1280. 10.1002/j.1532-2149.2012.00146.x 22528550

[39] Bacon KB, Harrison JK (2000) Chemokines and their receptors in neurobiology: perspectives in physiology and homeostasis. J Neuroimmunol 104: 92–97. 10.1016/S0165-5728(99)00266-0 10683519

[40] Bajetto A, Bonavia R, Barbero S, Florio T, Schettini G (2001) Chemokines and their receptors in the central nervous system. Front Neuroendocrinol 22: 147–184. 10.1006/frne.2001.0214 11456467

[41] Bajetto A, Bonavia R, Barbero S, Schettini G (2002) Characterization of chemokines and their receptors in the central nervous system: physiopathological implications. J Neurochem 82: 1311–1329. 10.1046/j.1471-4159.2002.01091.x 12354279

[42] Raghavendra V, Tanga FY, DeLeo JA (2004) Complete Freunds adjuvant-induced peripheral inflammation evokes glial activation and proinflammatory cytokine expression in the CNS. Eur J Neurosci 20: 467–473. 10.1111/j.1460-9568.2004.03514.x 15233755

[43] Sommer C (2003) Painful neuropathies. Curr Opin Neurol 16: 623–628. 10.1097/00019052-200310000-00009 14501847

[44] Tikka TM, Koistinaho JE (2001) Minocycline provides neuroprotection against N-methyl-D-aspartate neurotoxicity by inhibiting microglia. J Immunol 166: 7527–7533. 10.4049/jimmunol.166.12.7527 11390507

[45] Watkins LR, Maier SF (2002) Beyond neurons: evidence that immune and glial cells contribute to pathological pain states. Physiol Rev 82: 981–1011. 12270950 10.1152/physrev.00011.2002

[46] Wieseler-Frank J, Maier SF, Watkins LR (2004) Glial activation and pathological pain. Neurochem Int 45: 389–395. 10.1016/j.neuint.2003.09.009 15145553

[47] Lavon I, Goldberg I, Amit S, Landsman L, Jung S, et al (2000) High susceptibility to bacterial infection, but no liver dysfunction, in mice compromised for hepatocyte NF-kappaB activation. Nat Med 6: 573–577. 10.1038/75057 10802715

[48] O’Rielly DD, Loomis CW (2008) Spinal nerve ligation-induced activation of nuclear factor kappaB is facilitated by prostaglandins in the affected spinal cord and is a critical step in the development of mechanical allodynia. Neuroscience 155: 902–913. 10.1016/j.neuroscience.2008.04.077 18617333

